# Supplementary material for: Cell fate decision by a morphogen-transcription factor-chromatin modifier axis
Source: Nat Commun. 2024 Jul 29;15:6365. doi: 10.1038/s41467-024-50144-z (PMC11286941; doi:10.1038/s41467-024-50144-z)
Supplement: Supplementary file 1 — Supplementary information [file 41467_2024_50144_MOESM1_ESM.pdf]

Supplementary Figure. 1

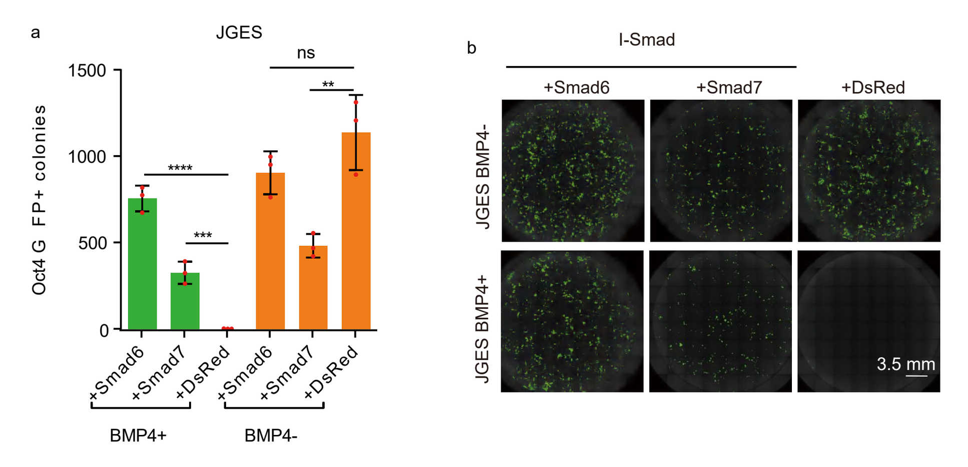

Supplementary Figure. 1

a. Bar plot for Oct4 GFP positive iPS colonies numbers of different gene over expression during JGES reprogramming under BMP4+ and BMP4- conditions at Day 7, data are mean ± s.d., two-sided, unpaired t test; n = 3 independent experiments, **p < 0.01, ***p < 0.001, ns: p>0.05.

b. Whole well screening photograph of Supplementary Figure 1a, scale bar = 3.5mm.

Supplementary Figure. 2

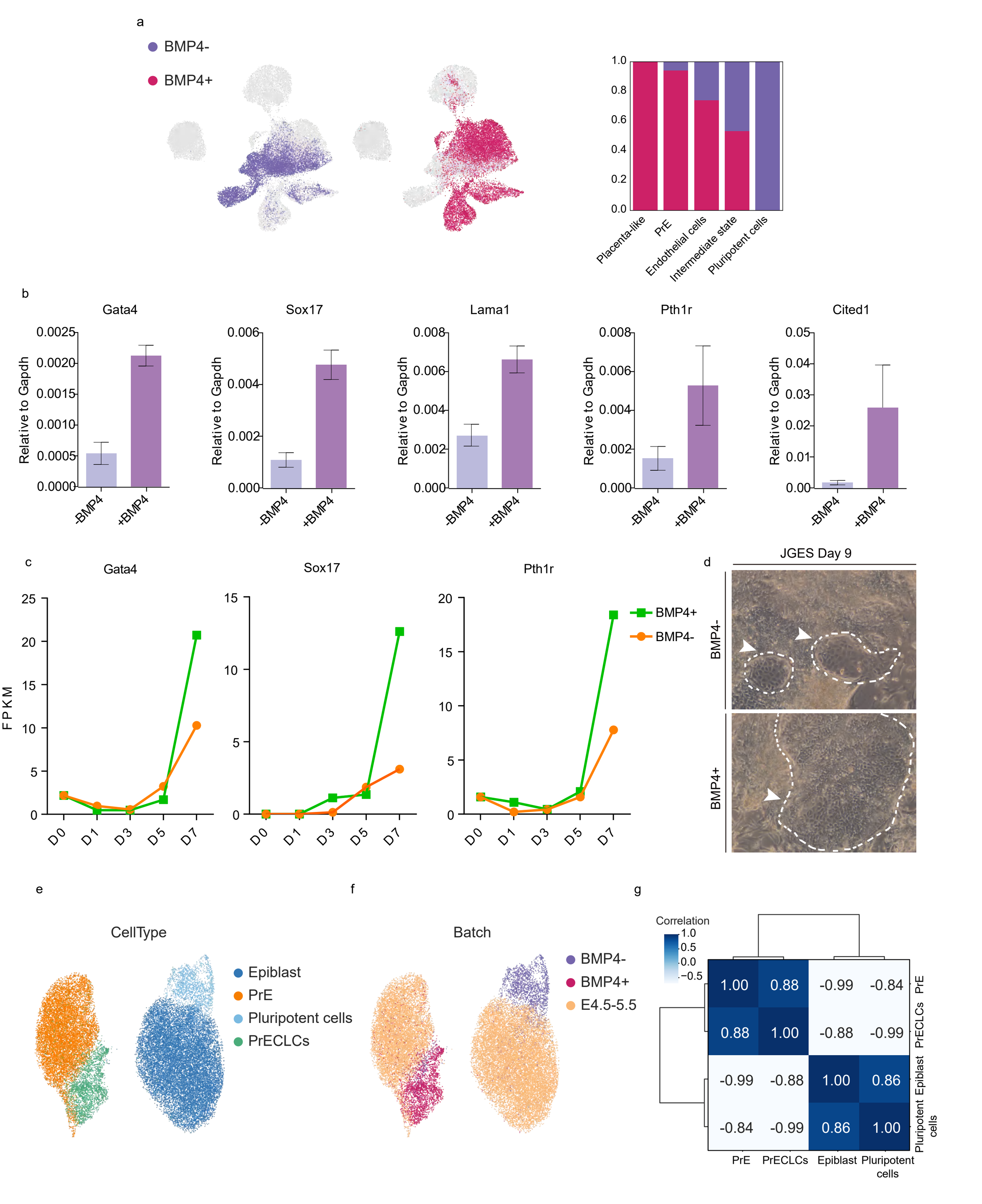

Supplementary Figure. 2

a. UMAP shows the cells distribution of BMP4+ and BMP4- conditions.

b: Stacked bar plots show the cell proportion in each cell type.

c-d. qPCR and Bulk RNA-seq results show the PrE marker genes expression at day 7 of JGES reprogramming under BMP4+ and BMP4- conditions.

e. Dotted line shows the PrECLCs morphology under BMP4+ and BMP4- treatment at day 9.

f-g. UMAP layout showing the integration of scRNA-seq data from this study and published E4.5-E5.5 mouse embryos[37].

h. Heatmap showing the Pearson correlation coefficient between PrECLCs and endodermal cell types in vivo. PrE, primitive endoderm; PaE, parietal endoderm; VE, visceral endoderm, the integration was performed by python package Harmony.

Supplementary Figure.3

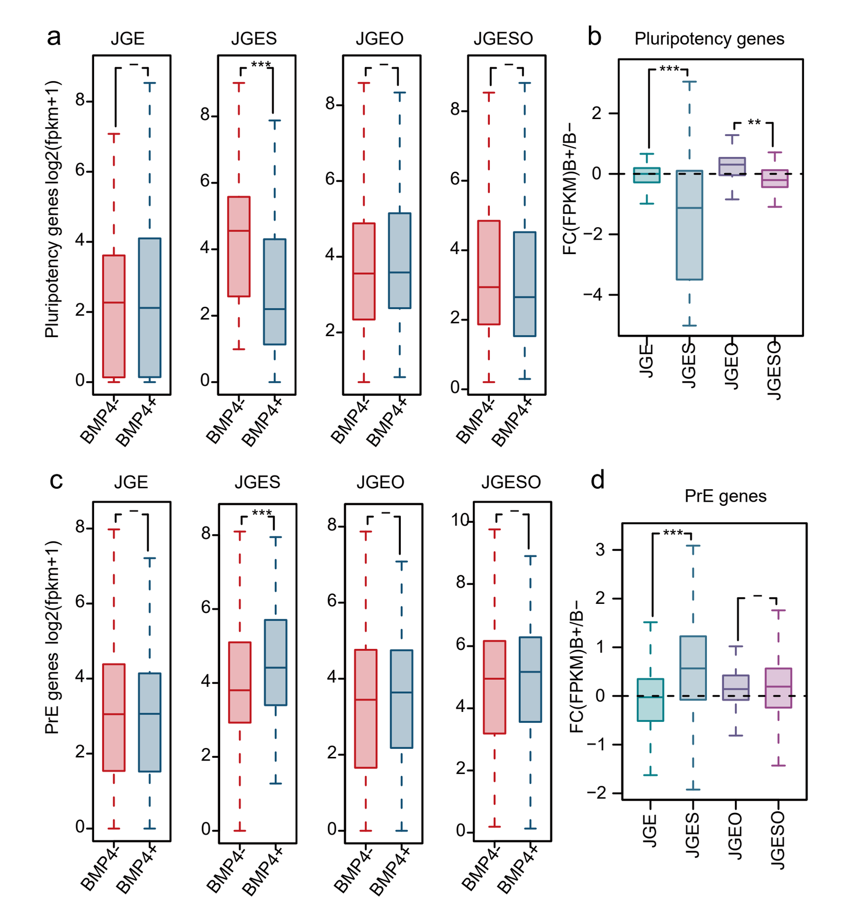

Supplementary Figure. 3

a. Box plot shows the expression levels of pluripolency genes between conditions with and without BMP4 treatment at day 7 during JGE, JGES, JGEO, JGESO reprogramming.

b. Box plot shows the fold changes in the expression of pluripotency genes between conditions with and without BMP4 treatment at day 7, during the JGE, JGES, JGEO, JGESO reprogramming, represented on a logarithmic scale.

c. Box plot shows the expression levels of PrE related genes between conditions with and without BMP4 treatment at day 7 during JGE, JGES, JGEO, JGESO reprogramming.

d. Box plot shows the fold changes in the expression of PrE related genes between conditions with and without BMP4 treatment at day 7, during the JGE, JGES, JGEO, JGESO reprogramming, represented on a logarithmic scale.

Supplementary Figure.4

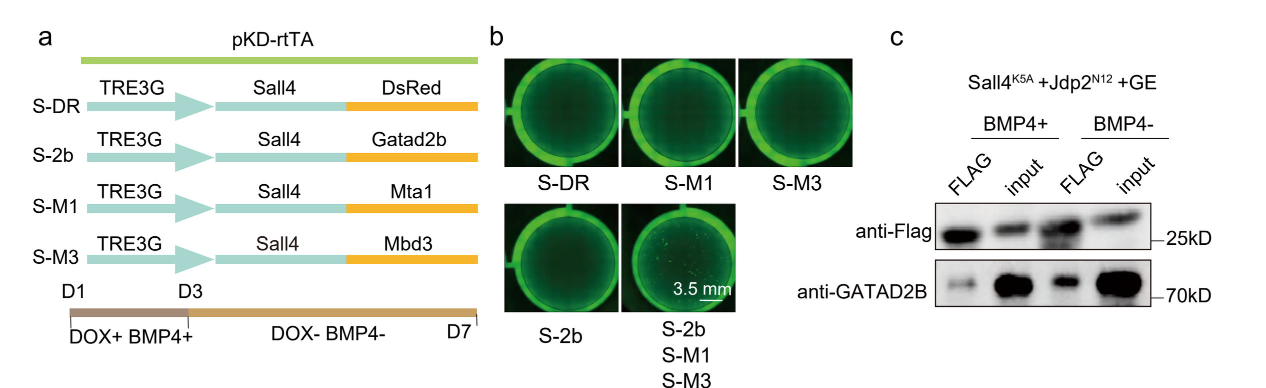

Supplementary Figure. 4

a. Schema chart shows the DOX-induced tet-on system applied in SALL4-MTA1, SALL4-MBD3, SALL4-GATAD2B fusion protein, DOX and BMP4 only added in the first 3 days .

b. Whole well screening Photograph of Figure 4d, scale bar = 3.5 mm

c. Western blots shows the results of SALL4-GATAD2B interaction under BMP4+ and BMP4- conditions at day 3 of Figure. 4e, FLAG beads were used to pull down Jdp2[N12] fusion with Flag tag, and interaction proteins from cell lysates.

Supplementary Figure.5

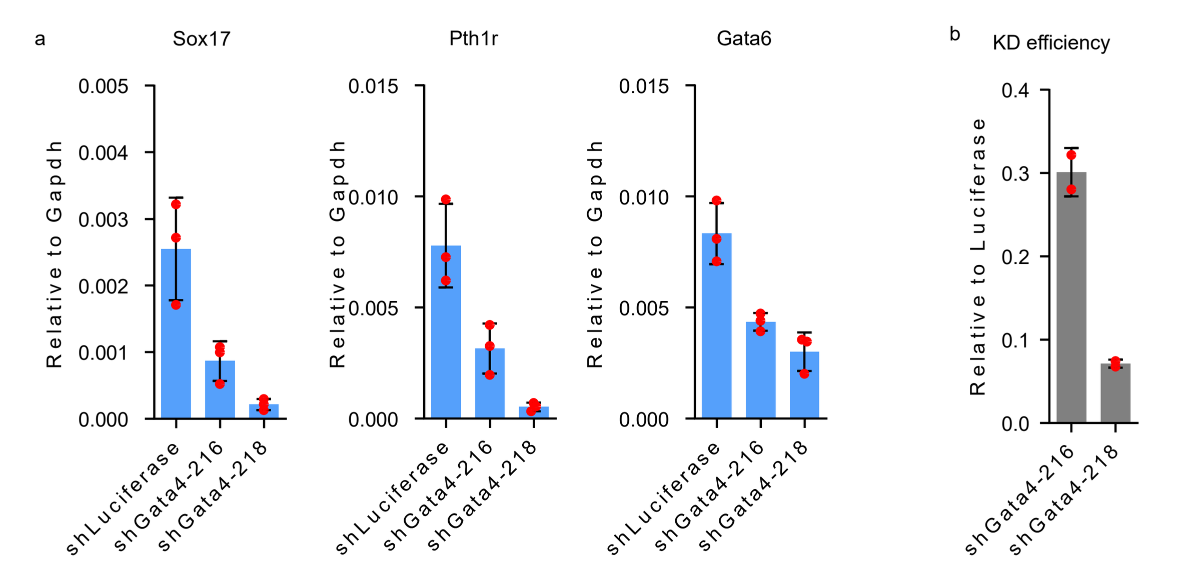

Supplementary Figure. 5

a. Histograms show the qPCR results of PrE gene relative expression level of every group, n = 3 independent experiments.

b. Histograms show the knock down efficiency of the two shRNAs of Gata4, n = 3 independent experiments.

Supplementary Figure.6

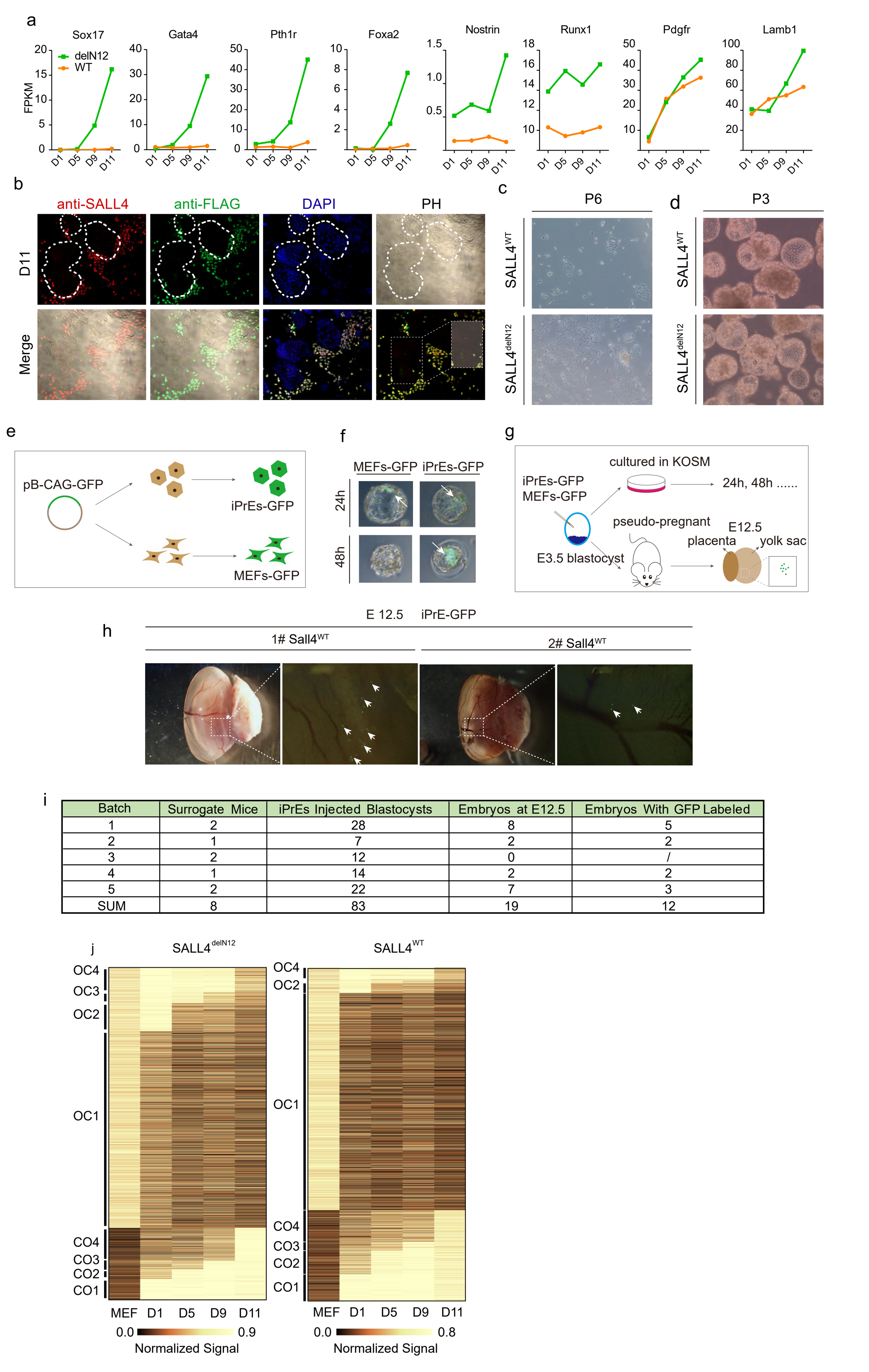

Supplementary Figure. 6

a. Bulk RNA-seq results show PrE marker genes expression level between Sall4[WT] and Sall4[delN12] at different time point.

b. Immunofluorescence staining shows the SALL4 expression in Sall4[delN12] induced PrE cells at day 11, dotted line indicates the iPrE clone, anti-SALL4 presents the endo- and exo- SALL4 expression, anti-FLAG presents the exo-SALL4 expression, cells colored in red alone in the square presents the endo-activation of SALL4.

c. Pictures show the 2D culture of iPrE induced by Sall4[WT] and Sall4[delN12] at passage 6.

d. Pictures show the 3D culture of iPrE induced by Sall4[WT] and Sall4[delN12] at passage 3

e. GFP sequence was constructed into pB vector with CAG promoter, and then transfected into iPrE cells induced by Sall4[WT] and MEF cells.

f. Blastocysts injected with iPrEs-GFP and MEFs-GFP culture in KOSM for 24h and 48h in vitro.

g. Flow chart shows the in vivo validation of developmental potency by blastocyte injection and transplantation of iPrE-GFP cells and MEFs-GFP cells.

h. Two pictures represent iPrE-GFP emerge at E12.5 yolk sac after transplantation.

i. Statistical data of Supplementary Figure 6h

j. Heatmaps show that the loci of open chromatin were arranged into groups depending upon the day of induction they changed from closed to open (CO) or open to closed (OC).

Supplementary Tables

Oligonucleotide Primers Table 1

Molecular Cloning	
pMX-Sall4-PF	agctagttaattaaggatccatgtcgaggcgcaagcaggc
pMX-Sall4-PR	ttgtagtccatggtacgcgtgctgacagcaatcttattttcctcc
PMX-Jdp2 PF	ggatctagctagttaattaaggatccatgatgcctgggcagatcc
PMX-Jdp2-PR	tactcgagactagtaagcttacgcgtctacttcttgtccagctgctcca
PMX-Glis1-PF	ggatctagctagttaattaaggatccatggcagaggcccgc
PMX-Glis1-PR	tttgatcaagaaagctgggtcttaggtgtctgtgtagatggagg
PMX-Esrrb-PF	gttaattaaggatccatgtcgtccgaagacaggcac
PMX-Esrrb-PR	gtccatggtacgcgtcaccttggcctccagcatc
PMX-Gata4-PF	gttaattaaggatccatgtaccaaagcctggccatg
PMX-Gata4-PR	gtccatggtacgcgtcgcggtgattatgtccccatg
PMX-Smad6-PF	ggatctagctagttaattaaggatccatgttcaggtctaaacgttcgg
PMX-Smad6-PR	tggtctttgtagtccatggtacgcgttctgtggttgttgagtaggatctcca
PMX-Smad7-PF	ggatctagctagttaattaaggatcccatgttcaggaccaaacgat
PMX-Smad7-PR	tggtctttgtagtccatggtacgcgtccggctgttgaagatgacctc
PMX-Sox17-PF	ggatctagctagttaattaaggatccatgagcagcccggatgc
PMX-Sox17-PR	catggtctttgtagtccatggtacgcgtaatgtcggggtagttgcaatagtagac
PMX-Pth1r-PF	ggatctagctagttaattaaggatccatggggaccgcccg
PMX-Pth1r-PR	tggtctttgtagtccatggtacgcgtcatgactgtttcccattcttcctg
PMX-Klf4-PF	ggatctagctagttaattaaggatccatgaggcagccacctgg
PMX-Klf4-PR	tggtctttgtagtccatggtacgcgtccttacacatgaagaggcacttt
PMX-Gata6-PF	ggatctagctagttaattaaggatccatggccttgactgacgg
PMX-Gata6-PR	tggtctttgtagtccatggtacgcgtggccagggccagagc
PMX-Pitx1-PF	ggatctagctagttaattaaggatccatggacgccttcaagggag
PMX-Pitx1-PR	tggtctttgtagtccatggtacgcgtgctgttgtactggcaagcg
PMX-Foxa2-PF	ggatctagctagttaattaaggatcc atgcactcggcttccagtatgctgg
PMX-Foxa2-PR	tggtctttgtagtccatggtacgcgtggatgagttcataataggcctggagt

Oligonucleotide Primers Table 2

qPCR primers	
Gata4-qPF	CCCTACCCAGCCTACATGG
Gata4-qPR	ACATATCGAGATTGGGGTGTCT
Gata6-qPF	TTGCTCCGGTAACAGCAGTG
Gata6-qPR	GTGGTCGCTTGTGTAGAAGGA
Sox17-qPF	GATGCGGGATACGCCAGTG
Sox17-qPR	CCACCTCGCCTTTCACCTTTA
Lama1-qPF	ACTATGCCGTCAGCGATACAG
Lama1-qPR	GGCACCAGCTTTGAATAATACGA
Pth1r-qPF	CAGGCGCAATGTGACAAGC
Pth1r-qPR	TTTCCCGGTGCCTTCTCTTTC
Cited1-qPF	AACCTTGGAGTGAAGGATCGC
Cited1-qPR	GTAGGAGAGCCTATTGGAGATGT
Dab2-qPF	CCCCTGAACGGTGATACTGAT
Dab2-qPR	AAGTCCTGCTTTACGCCATTC
Gapdh-qPF	ACCTGCCAAGTATGATGAC
Gapdh-qPFR	GGGAGTTGCTGTTGAAGT

shRNA Sequence Table

Gata4 KD shRNA	
216	AGCCCAAGAACCTGAATAAAT
218	GAAGGCAGAGAGTGTGTCAAT
