# Supplementary material for: Cell fate decision by a morphogen-transcription factor-chromatin modifier axis
Source: Nat Commun. 2024 Jul 29;15:6365. doi: 10.1038/s41467-024-50144-z (PMC11286941; doi:10.1038/s41467-024-50144-z)
Supplement: Supplementary file 2 — Reporting Summary [file 41467_2024_50144_MOESM2_ESM.pdf]

nature portfolio

Corresponding author(s): NCOMMS-23-44851A

Last updated by author(s): Jun 9, 2024

Reporting Summary

Nature Portfolio wishes to improve the reproducibility of the work that we publish. This form provides structure for consistency and transparency in reporting. For further information on Nature Portfolio policies, see our Editorial Policies and the Editorial Policy Checklist.

Statistics

For all statistical analyses, confirm that the following items are present in the figure legend, table legend, main text, or Methods section.

n/a	Confirmed	
☐	☒	The exact sample size (n) for each experimental group/condition, given as a discrete number and unit of measurement
☐	☒	A statement on whether measurements were taken from distinct samples or whether the same sample was measured repeatedly
☐	☒	The statistical test(s) used AND whether they are one- or two-sided<br>*Only common tests should be described solely by name; describe more complex techniques in the Methods section.*
☒	☐	A description of all covariates tested
☒	☐	A description of any assumptions or corrections, such as tests of normality and adjustment for multiple comparisons
☐	☒	A full description of the statistical parameters including central tendency (e.g. means) or other basic estimates (e.g. regression coefficient) AND variation (e.g. standard deviation) or associated estimates of uncertainty (e.g. confidence intervals)
☐	☒	For null hypothesis testing, the test statistic (e.g. F, t, r) with confidence intervals, effect sizes, degrees of freedom and P value noted<br>*Give P values as exact values whenever suitable.*
☒	☐	For Bayesian analysis, information on the choice of priors and Markov chain Monte Carlo settings
☒	☐	For hierarchical and complex designs, identification of the appropriate level for tests and full reporting of outcomes
☒	☐	Estimates of effect sizes (e.g. Cohen's d, Pearson's r), indicating how they were calculated

Our web collection on statistics for biologists contains articles on many of the points above.

Software and code

Policy information about availability of computer code

Data collection	Bulk RNA-seq data are collected at home by using the specific commercial kit(Vazyme, NR611). scRNA-seq data are collected at home by using the specific commercial kit(Chromium Single Cell 3' Reagent Kits v3.1). ATAC-seq data are collected at home by using the specific commercial kit(Vazyme, TD501); Cut&Tag data are collected at home by using the specific commercial kit(Vazyme, TD903)
Data analysis	Processing of scRNA-sequencing data : The FASTQ files of single-cell libraries generated from Illumina NovaSeq. The clean FASTQ files were aligned to the Mm10 genome with mouse gene annotation of Gencode vM21 version by STARsolo function of STAR (2.7.6a)48. Low-quality cells were filtered out by the number of unique molecular identifiers (UMIs) and total counts following the pipeline of python package Scanpy49. Gene Regulatory Network (GRN) analysis: We performed GRN analysis using pySCENIC50. We obtained regulon score for all cells of each transcription factor . The importance of transcription factors for each cell types was ordered by normalized enrichment score.<br><br>RNA-seq and data analysis : The RNA-seq reads were trimmed using Trim Galore (v0.6.4) and then mapped to the mm10 reference genome with HISAT2 (v2.2.1), and StringTie (v2.2.1) was used to quantify the transcription level of each gene in each sample into FPKM (Fragments per kilobase of exon model per million mapped reads). GFOLD (v1.1.4) was used to perform differential expression analysis between conditions. The differentially expressed genes were identified with gfold value > 0.5.<br>ATAC-seq and data analysis : The ATAC-seq reads were trimmed by Trim Galore (v0.6.4) and then mapped to the mm10 reference genome using bowtie2 (v2.4.5)56, and SAMtools (v1.16.1)57 was used to remove the unpaired, low sequencing quality (mapq<30) and the mitochondrial DNA mapped reads in the total mapped reads. The reads that length less than 50 base pairs (bp) were isolated for subsequent analysis. In order to make the data comparable between different sequencing depths, the signals were normalized to one million reads for each sample, and the value were further compressed into a binary format (bigWig) for downstream analysis and data visualization. Peak calling was performed using MACS (v1.4.2)58 with parameters as follows: -g mm --keep-dup all --nomodel --shiftsize 25.

Cut&Tag and data analysis：The CUT&Tag reads were trimmed by Trim Galore (v0.6.4) and then mapped to the mm10 reference genome using bowtie2 (v2.4.5). SAMtools (v1.16.1) was used to remove the repetitive, low sequencing quality (mapq<30) and the mitochondrial DNA mapped reads in the total mapped reads. The value were further compressed into a binary format for downstream analysis and data visualization. Replicates were merged using samtools merge and then peak calling was performed using MACS (v1.4.2) with parameters as follows: -g mm --keep-dup 1 --nomodel --shiftsize 73. The signals were normalized to one million reads for each sample. Promoters were defined as regions ± 2 kb around transcription start sites (TSSs) of genes.
Motif analysis: Motif scans were performed using HOMER (v4.11.1)59 against the genome sequence of the given ATAC-seq peaks covered regions (summits ± 25 bp) with the following parameters: -size given -mask. HOMER used hypergeometric test to determine the motif enrichment and also test the similarity between the motif we identified to known factors. Motifs which have p-value < 10-5 and enrichment score > 3 are presented in the plot.
Gene Ontology analysis: Functional annotation was performed using the clusterProfiler (v4.6.2)60. Gene Ontology terms for each functional cluster were summarized to a representative term, and adjusted p-values were plotted to show the significance.

For manuscripts utilizing custom algorithms or software that are central to the research but not yet described in published literature, software must be made available to editors and reviewers. We strongly encourage code deposition in a community repository (e.g. GitHub). See the Nature Portfolio guidelines for submitting code & software for further information.

Data

Policy information about availability of data

All manuscripts must include a data availability statement. This statement should provide the following information, where applicable:

- Accession codes, unique identifiers, or web links for publicly available datasets
- A description of any restrictions on data availability
- For clinical datasets or third party data, please ensure that the statement adheres to our policy

Bulk and scRNA-seq data,   ATAC-seq data, Cut&Tag data from this study have been deposited into the Gene Expression Omnibus (GEO) database under the accession number GSE242851.

Research involving human participants, their data, or biological material

Policy information about studies with human participants or human data. See also policy information about sex, gender (identity/presentation), and sexual orientation and race, ethnicity and racism.

Reporting on sex and gender	*Use the terms sex (biological attribute) and gender (shaped by social and cultural circumstances) carefully in order to avoid confusing both terms. Indicate if findings apply to only one sex or gender; describe whether sex and gender were considered in study design; whether sex and/or gender was determined based on self-reporting or assigned and methods used. Provide in the source data disaggregated sex and gender data, where this information has been collected, and if consent has been obtained for sharing of individual-level data; provide overall numbers in this Reporting Summary. Please state if this information has not been collected. Report sex- and gender-based analyses where performed, justify reasons for lack of sex- and gender-based analysis.*
Reporting on race, ethnicity, or other socially relevant groupings	*Please specify the socially constructed or socially relevant categorization variable(s) used in your manuscript and explain why they were used. Please note that such variables should not be used as proxies for other socially constructed/relevant variables (for example, race or ethnicity should not be used as a proxy for socioeconomic status). Provide clear definitions of the relevant terms used, how they were provided (by the participants/respondents, the researchers, or third parties), and the method(s) used to classify people into the different categories (e.g. self-report, census or administrative data, social media data, etc.) Please provide details about how you controlled for confounding variables in your analyses.*
Population characteristics	*Describe the covariate-relevant population characteristics of the human research participants (e.g. age, genotypic information, past and current diagnosis and treatment categories). If you filled out the behavioural & social sciences study design questions and have nothing to add here, write "See above."*
Recruitment	*Describe how participants were recruited. Outline any potential self-selection bias or other biases that may be present and how these are likely to impact results.*
Ethics oversight	*Identify the organization(s) that approved the study protocol.*

Note that full information on the approval of the study protocol must also be provided in the manuscript.

Field-specific reporting

Please select the one below that is the best fit for your research. If you are not sure, read the appropriate sections before making your selection.

[x] Life sciences   [ ] Behavioural & social sciences   [ ] Ecological, evolutionary & environmental sciences

For a reference copy of the document with all sections, see nature.com/documents/nr-reporting-summary-flat.pdf

Life sciences study design

All studies must disclose on these points even when the disclosure is negative.

Sample size	The sample size for each figure in this study has been described in the figure legends relevantly.
Data exclusions	No data were exclude from the analysis.
Replication	Data are presented as mean±s.d. as indicated in the Fig legends. For all the main figures from which the significance were assessed, at least 2 biological replication experiment were preformed.
Randomization	The authors thought there was no relevant for randomization to our study.
Blinding	The investigates were blinded to group allocation during data collection and analysis.

Behavioural & social sciences study design

All studies must disclose on these points even when the disclosure is negative.

Study description	*Briefly describe the study type including whether data are quantitative, qualitative, or mixed-methods (e.g. qualitative cross-sectional, quantitative experimental, mixed-methods case study).*
Research sample	*State the research sample (e.g. Harvard university undergraduates, villagers in rural India) and provide relevant demographic information (e.g. age, sex) and indicate whether the sample is representative. Provide a rationale for the study sample chosen. For studies involving existing datasets, please describe the dataset and source.*
Sampling strategy	*Describe the sampling procedure (e.g. random, snowball, stratified, convenience). Describe the statistical methods that were used to predetermine sample size OR if no sample-size calculation was performed, describe how sample sizes were chosen and provide a rationale for why these sample sizes are sufficient. For qualitative data, please indicate whether data saturation was considered, and what criteria were used to decide that no further sampling was needed.*
Data collection	*Provide details about the data collection procedure, including the instruments or devices used to record the data (e.g. pen and paper, computer, eye tracker, video or audio equipment) whether anyone was present besides the participant(s) and the researcher, and whether the researcher was blind to experimental condition and/or the study hypothesis during data collection.*
Timing	*Indicate the start and stop dates of data collection. If there is a gap between collection periods, state the dates for each sample cohort.*
Data exclusions	*If no data were excluded from the analyses, state so OR if data were excluded, provide the exact number of exclusions and the rationale behind them, indicating whether exclusion criteria were pre-established.*
Non-participation	*State how many participants dropped out/declined participation and the reason(s) given OR provide response rate OR state that no participants dropped out/declined participation.*
Randomization	*If participants were not allocated into experimental groups, state so OR describe how participants were allocated to groups, and if allocation was not random, describe how covariates were controlled.*

Ecological, evolutionary & environmental sciences study design

All studies must disclose on these points even when the disclosure is negative.

Study description	*Briefly describe the study. For quantitative data include treatment factors and interactions, design structure (e.g. factorial, nested, hierarchical), nature and number of experimental units and replicates.*
Research sample	*Describe the research sample (e.g. a group of tagged Passer domesticus, all Stenocereus thurberi within Organ Pipe Cactus National Monument), and provide a rationale for the sample choice. When relevant, describe the organism taxa, source, sex, age range and any manipulations. State what population the sample is meant to represent when applicable. For studies involving existing datasets, describe the data and its source.*
Sampling strategy	*Note the sampling procedure. Describe the statistical methods that were used to predetermine sample size OR if no sample-size calculation was performed, describe how sample sizes were chosen and provide a rationale for why these sample sizes are sufficient.*
Data collection	*Describe the data collection procedure, including who recorded the data and how.*
Timing and spatial scale	*Indicate the start and stop dates of data collection, noting the frequency and periodicity of sampling and providing a rationale for these choices. If there is a gap between collection periods, state the dates for each sample cohort. Specify the spatial scale from which the data are taken*

Data exclusions	*If no data were excluded from the analyses, state so OR if data were excluded, describe the exclusions and the rationale behind them, indicating whether exclusion criteria were pre-established.*
Reproducibility	*Describe the measures taken to verify the reproducibility of experimental findings. For each experiment, note whether any attempts to repeat the experiment failed OR state that all attempts to repeat the experiment were successful.*
Randomization	*Describe how samples/organisms/participants were allocated into groups. If allocation was not random, describe how covariates were controlled. If this is not relevant to your study, explain why.*
Blinding	*Describe the extent of blinding used during data acquisition and analysis. If blinding was not possible, describe why OR explain why blinding was not relevant to your study.*

Did the study involve field work? ☐ Yes ☐ No

Field work, collection and transport

Field conditions	*Describe the study conditions for field work, providing relevant parameters (e.g. temperature, rainfall).*
Location	*State the location of the sampling or experiment, providing relevant parameters (e.g. latitude and longitude, elevation, water depth).*
Access & import/export	*Describe the efforts you have made to access habitats and to collect and import/export your samples in a responsible manner and in compliance with local, national and international laws, noting any permits that were obtained (give the name of the issuing authority, the date of issue, and any identifying information).*
Disturbance	*Describe any disturbance caused by the study and how it was minimized.*

Reporting for specific materials, systems and methods

We require information from authors about some types of materials, experimental systems and methods used in many studies. Here, indicate whether each material, system or method listed is relevant to your study. If you are not sure if a list item applies to your research, read the appropriate section before selecting a response.

Materials & experimental systems

n/a	Involved in the study	
☐	☒	Antibodies
☐	☒	Eukaryotic cell lines
☒	☐	Palaeontology and archaeology
☐	☒	Animals and other organisms
☒	☐	Clinical data
☒	☐	Dual use research of concern
☒	☐	Plants

Methods

n/a	Involved in the study	
☐	☒	ChIP-seq
☐	☒	Flow cytometry
☒	☐	MRI-based neuroimaging

Antibodies

Antibodies used	NuRD Complex Antibody Sampler Kit (CST,8349T), Anti GATAD2B (Abcam, ab224391), anti RBBP4(Novusbio NB500-123), anti-FLAG (Sigma Aldrich, F1804 1:1000), anti SALL4 (Abcam, ab29112), anti-FLAG beads, DYKDDDDK (Themo Fisher, A36797) anti-GATA4 (Santa Cruz Biotechnology, sc-25310), anti-LAMA1 (Abcam,ab11575) were used.
Validation	These antibodies were validAbated in CiteAb website

Eukaryotic cell lines

Policy information about cell lines and Sex and Gender in Research

Cell line source(s)	OG2 MEF cells were derived from E13.5 mouse generated by mating homozygous Oct4-GFP transgenic allele-carrying mice (CBA/CaJ X C57BL/6J) with 129/Sv female mice.
Authentication	MEF cell lines were characteristic in home by chimeric mice test in home.
Mycoplasma contamination	All of the cell lines have been confirmed as mycoplasma contamination free with the kit from lonza(LT07-318)
Commonly misidentified lines (See ICLAC register)	no

Palaeontology and Archaeology

Specimen provenance	*Provide provenance information for specimens and describe permits that were obtained for the work (including the name of the issuing authority, the date of issue, and any identifying information). Permits should encompass collection and, where applicable, export.*
Specimen deposition	*Indicate where the specimens have been deposited to permit free access by other researchers.*
Dating methods	*If new dates are provided, describe how they were obtained (e.g. collection, storage, sample pretreatment and measurement), where they were obtained (i.e. lab name), the calibration program and the protocol for quality assurance OR state that no new dates are provided.*

☐ Tick this box to confirm that the raw and calibrated dates are available in the paper or in Supplementary Information.

Ethics oversight	*Identify the organization(s) that approved or provided guidance on the study protocol, OR state that no ethical approval or guidance was required and explain why not.*

Note that full information on the approval of the study protocol must also be provided in the manuscript.

Animals and other research organisms

Policy information about studies involving animals; ARRIVE guidelines recommended for reporting animal research, and Sex and Gender in Research

Laboratory animals	Oct4-GFP (OG2) transgenic-allele-carrying mice (CBA/CaJ×C57BL/6J) were purchased from The Jackson Laboratory, (Mouse strain datasheet: 004654). 129Sv/Jae and ICR mice were purchased from Beijing Vital River Laboratory. Animals were individually housed under a 12h light/dark cycle and provided with food and water ad libitum, ambient temperature is about 20℃-26℃ and humidity is about 40%-70%. OG2 mice and 129Sv/Jae mice were used to generate E13.5 mouse embryo fibroblast (MEFs), ICR mice were used to apply donor blastocyst and pseudo-pregnant mice.
Wild animals	no
Reporting on sex	no
Field-collected samples	no
Ethics oversight	Our studies followed the guidelines for the Care and Use of Laboratory Animals of the National Institutes of Health, and the protocols were approved by the Committee on the Ethics of Animal Experiments at the Guangzhou Institutes of Biomedicine and Health.

Note that full information on the approval of the study protocol must also be provided in the manuscript.

Clinical data

Policy information about clinical studies

All manuscripts should comply with the ICMJEguidelines for publication of clinical research and a completedCONSORT checklist must be included with all submissions.

Clinical trial registration	*Provide the trial registration number from ClinicalTrials.gov or an equivalent agency.*
Study protocol	*Note where the full trial protocol can be accessed OR if not available, explain why.*
Data collection	*Describe the settings and locales of data collection, noting the time periods of recruitment and data collection.*
Outcomes	*Describe how you pre-defined primary and secondary outcome measures and how you assessed these measures.*

Dual use research of concern

Policy information about dual use research of concern

Hazards

Could the accidental, deliberate or reckless misuse of agents or technologies generated in the work, or the application of information presented in the manuscript, pose a threat to:

No | Yes

- [ ] [ ] Public health
- [ ] [ ] National security
- [ ] [ ] Crops and/or livestock
- [ ] [ ] Ecosystems
- [ ] [ ] Any other significant area

Experiments of concern

Does the work involve any of these experiments of concern:

No | Yes

- [ ] [ ] Demonstrate how to render a vaccine ineffective
- [ ] [ ] Confer resistance to therapeutically useful antibiotics or antiviral agents
- [ ] [ ] Enhance the virulence of a pathogen or render a nonpathogen virulent
- [ ] [ ] Increase transmissibility of a pathogen
- [ ] [ ] Alter the host range of a pathogen
- [ ] [ ] Enable evasion of diagnostic/detection modalities
- [ ] [ ] Enable the weaponization of a biological agent or toxin
- [ ] [ ] Any other potentially harmful combination of experiments and agents

Plants

Seed stocks

Report on the source of all seed stocks or other plant material used. If applicable, state the seed stock centre and catalogue number. If plant specimens were collected from the field, describe the collection location, date and sampling procedures.

Novel plant genotypes

Describe the methods by which all novel plant genotypes were produced. This includes those generated by transgenic approaches, gene editing, chemical/radiation-based mutagenesis and hybridization. For transgenic lines, describe the transformation method, the number of independent lines analyzed and the generation upon which experiments were performed. For gene-edited lines, describe the editor used, the endogenous sequence targeted for editing, the targeting guide RNA sequence (if applicable) and how the editor was applied.

Authentication

Describe any authentication procedures for each seed stock used or novel genotype generated. Describe any experiments used to assess the effect of a mutation and, where applicable, how potential secondary effects (e.g. second site T-DNA insertions, mosiacism, off-target gene editing) were examined.

ChIP-seq

Data deposition

- [x] Confirm that both raw and final processed data have been deposited in a public database such as GEO.
- [x] Confirm that you have deposited or provided access to graph files (e.g. BED files) for the called peaks.

Data access links
May remain private before publication.

https://www.ncbi.nlm.nih.gov/geo/query/acc.cgi?acc=GSE242851
review token: mvsjeswgrlehfkn

Files in database submission

CUT&Tag(H3K27ac_SALL4-delN12 Day6)
CUT&Tag(SALL4_SALL4-delN12 Day6)
CUT&Tag(H3K27ac_SALL4-WT Day6)
CUT&Tag(SALL4_SALL4-WT Day6)

Genome browser session
(e.g. UCSC)

no longer applicable

Methodology

Replicates

Describe the experimental replicates, specifying number, type and replicate agreement.

Sequencing depth

SampleName Layout TotalPairs UnmappedPairs UniqMappedPairs MultiMappedPairs DiscordantlyAlignedPairs UnpairedReads UnmappedReads UniqMappedReads MultiMappedReads OverallAlignmentRate UniqAlignmentRate
WT_SALL4 pair 34586478 2143607 26977201 5465670 873148 2540918 1628868 568945 343105 97.65% 78.00%
WT_H3K27ac pair 34665302 4986517 27061695 2617090 3364432 3244170 1562301 995887 685982 97.75% 78.07%
N12_SALL4 pair 33996163 3242959 27083777 3669427 1724361 3037196 1686143 891145 459908 97.52% 79.67%
N12_H3K27ac pair 42120511 2665585 36480350 2974576 1229919 2871332 1749036 845860 276436 97.92% 86.61%

Antibodies	anti SALL4 (Abcam, ab29112), anti-H3K27ac (Abcam ab4729), were used.
Peak calling parameters	macs14 callpeak -g mm -p 1e-5 --keep-dup 1 --nomodel --shiftsize 73 -t ${sample}.bam -n ${sample}
Data quality	SAMtools (v1.16.1) was used to remove the repetitive, low sequencing quality (mapq<30) and the mitochondrial DNA mapped reads in the total mapped reads.
Software	The CUT&Tag reads were trimmed by Trim Galore (v0.6.4) and then mapped to the mm10 reference genome using bowtie2 (v2.4.5).

Flow Cytometry

Plots

Confirm that:

☒ The axis labels state the marker and fluorochrome used (e.g. CD4-FITC).

☒ The axis scales are clearly visible. Include numbers along axes only for bottom left plot of group (a 'group' is an analysis of identical markers).

☒ All plots are contour plots with outliers or pseudocolor plots.

☒ A numerical value for number of cells or percentage (with statistics) is provided.

Methodology

Sample preparation	Cells were dissociated into single cells using 0.25% trypsin-EDTA and collected using centrifugation. After washing once with PBS, the cell pellet was resuspended with PBS containing 0.1% BSA, followed by filtration using a cell strainer (BD Biosciences) to remove large clumps of cells.
Instrument	Accuri C6 flow cytometer (BD Biosciences).
Software	FlowJo v.7.6.1.
Cell population abundance	20000 cells collected per sample
Gating strategy	The GFP fluorescence intensity was detected in the FITC channel.

☒ Tick this box to confirm that a figure exemplifying the gating strategy is provided in the Supplementary Information.

Magnetic resonance imaging

Experimental design

Design type	*Indicate task or resting state; event-related or block design.*
Design specifications	*Specify the number of blocks, trials or experimental units per session and/or subject, and specify the length of each trial or block (if trials are blocked) and interval between trials.*
Behavioral performance measures	*State number and/or type of variables recorded (e.g. correct button press, response time) and what statistics were used to establish that the subjects were performing the task as expected (e.g. mean, range, and/or standard deviation across subjects).*

Acquisition

Imaging type(s)	*Specify: functional, structural, diffusion, perfusion.*
Field strength	*Specify in Tesla*
Sequence & imaging parameters	*Specify the pulse sequence type (gradient echo, spin echo, etc.), imaging type (EPI, spiral, etc.), field of view, matrix size, slice thickness, orientation and TE/TR/flip angle.*
Area of acquisition	*State whether a whole brain scan was used OR define the area of acquisition, describing how the region was determined.*

Diffusion MRI ☐ Used ☐ Not used

Preprocessing

Preprocessing software	*Provide detail on software version and revision number and on specific parameters (model/functions, brain extraction, segmentation, smoothing kernel size, etc.).*

Normalization | *If data were normalized/standardized, describe the approach(es): specify linear or non-linear and define image types used for transformation OR indicate that data were not normalized and explain rationale for lack of normalization.*

Normalization template | *Describe the template used for normalization/transformation, specifying subject space or group standardized space (e.g. original Talairach, MNI305, ICBM152) OR indicate that the data were not normalized.*

Noise and artifact removal | *Describe your procedure(s) for artifact and structured noise removal, specifying motion parameters, tissue signals and physiological signals (heart rate, respiration).*

Volume censoring | *Define your software and/or method and criteria for volume censoring, and state the extent of such censoring.*

Statistical modeling & inference

Model type and settings | *Specify type (mass univariate, multivariate, RSA, predictive, etc.) and describe essential details of the model at the first and second levels (e.g. fixed, random or mixed effects; drift or auto-correlation).*

Effect(s) tested | *Define precise effect in terms of the task or stimulus conditions instead of psychological concepts and indicate whether ANOVA or factorial designs were used.*

Specify type of analysis: ☐ Whole brain ☐ ROI-based ☐ Both

Statistic type for inference | *Specify voxel-wise or cluster-wise and report all relevant parameters for cluster-wise methods.*

(See Eklund et al. 2016)

Correction | *Describe the type of correction and how it is obtained for multiple comparisons (e.g. FWE, FDR, permutation or Monte Carlo).*

Models & analysis

n/a	Involved in the study
☐	☐ Functional and/or effective connectivity
☐	☐ Graph analysis
☐	☐ Multivariate modeling or predictive analysis

Functional and/or effective connectivity | *Report the measures of dependence used and the model details (e.g. Pearson correlation, partial correlation, mutual information).*

Graph analysis | *Report the dependent variable and connectivity measure, specifying weighted graph or binarized graph, subject- or group-level, and the global and/or node summaries used (e.g. clustering coefficient, efficiency, etc.).*

Multivariate modeling and predictive analysis | *Specify independent variables, features extraction and dimension reduction, model, training and evaluation metrics.*
